# Habitat imaging based on DCE-MRI for differentiating luminal and non-luminal subtypes of breast cancer: a two-center study

**DOI:** 10.3389/fonc.2026.1899349

**Published:** 2026-09-07

**Authors:** Zhang Qing, Qin Xiao-Tao, Zhou Xia, Zhang Xiu-Lan, Peng Jie, Li Yun, Wang Wei-Bing

**Affiliations:** 1 Department of Radiology, The First Affiliated Hospital of Yangtze University, Hubei, China; 2 Department of Radiology, Zhuzhou Central Hospital, Hunan, China

**Keywords:** breast cancer, heterogeneity, luminal, magnetic resonance, radiomics

## Abstract

**Objective:**

To explore the application value of habitat imaging based on dynamic contrast-enhanced magnetic resonance imaging (DCE-MRI) for differentiating luminal and non-luminal subtypes of breast cancer (BC).

**Methods:**

Retrospective data from 396 BC patients across two centers were collected. The data from Center 1 were split into a training set of 220 patients and an internal validation set of 56 patients, while Center 2 provided an external test set of 120 patients. Multivariable analysis was performed to identify independent risk factors for developing the clinical model. K-means algorithm was used to perform clustering on DCE-MRI. After feature extraction and selection, eight machine learning algorithms were utilized to build traditional radiomics model, habitat model, and clinical model. A stacking fusion strategy was employed to integrate the traditional radiomics model, habitat model, and clinical model for identifying luminal and non-luminal subtypes patients. Model performance was evaluated using receiver operating characteristic (ROC) curves, calibration curve and decision curve analysis (DCA). Shapley Additive Explanations (SHAP) was performed for model interpretability.

**Results:**

For the discrimination of breast cancer luminal and non-luminal subtypes, the stacking model yielded the largest area under the curve value (AUC = 0.840), followed by the habitat model and the conventional radiomics model (AUC = 0.830, AUC = 0.805, respectively), all of which were significantly better than the clinical model (p < 0.05, respectively). Calibration curves showed good calibration of the stacking model, and decision curves confirmed its favorable net clinical benefit. SHAP revealed habitat-LGBM in the stacking model with their contribution being particularly prominent.

**Conclusion:**

Habitat imaging exhibits promising potential to differentiate luminal and non-luminal breast cancer subtypes. The stacking model integrating habitat-LGBM, traditional radiomics-LGBM and clinical-XGBoost may provide favorable predictive performance.

## 1 Introduction

Breast cancer (BC) causes the leading cancer-related death in women worldwide (1). It is a biologically diverse disease with identified molecular subgroups associated with varying therapies and prognoses (2). According to the expression of the estrogen receptor (ER), progesterone receptor (PR), BC can be classified into luminal and non-luminal types (3). Overall, luminal cancers respond well to endocrine therapies and typically have a favorable prognosis. Non-luminal BC is more aggressive and has a higher likelihood of tumor recurrence and metastasis (4). Therefore, accurate preoperative assessment of molecular subtypes of BC is very important for formulating individualized treatment strategies and improving prognosis. Currently, the preoperative evaluation of molecular subtypes primarily depends on immunohistochemistry (IHC) results obtained from core needle biopsy (CNB). However, CNB is time-consuming, poorly tolerated for repeated assessment, and prone to sampling bias caused by tumor heterogeneity. Subtyping based on clinicopathological features alone remains limited (5, 6), creating a need for more reliable imaging-based tools.

MR-based radiomics extracts a wide range of quantitative features that offer a thorough characterization of tumors (7). However, this computational approach is applicable to the entire tumor and assumes that the heterogeneity is uniformly present, thereby over-looking regional phenotypic variations within the tumor. Habitat imaging is an image processing technique that divides the entire lesion into multiple heterogeneous or homogeneous subregions based on differences in histopathology and molecular biology. Compared with conventional radiomics, this method improves the quantification of intratumoral heterogeneity and enables noninvasive evaluation of internal tumor heterogeneity (8, 9). Accumulating evidence has consistently validated MRI radiomics for the accurate differentiation of luminal and non-luminal breast cancer molecular subtypes (10–12). Up to date, however, the values of MRI-based habitat radiomics in Luminal or non-luminal molecular subtype of BC have not been investigated.

We hypothesized that genetic and structural heterogeneity in luminal breast cancer leads to greater gray-level variations and distinct textural features on DCE-MRI, detectable by radiomics analysis. This study aimed to explore the feasibility of DCE-MRI-based habitat radiomics in distinguishing luminal from non-luminal breast cancer, comparison with conventional radiomics, and to construct a comprehensive predictive model by integrating habitat radiomics, conventional radiomics, and clinical features.

## 2 Materials and methods

### 2.1 Study population

This retrospective study was approved by the Medical Ethics Committee of the First People’s Hospital of Yangtze University (Approval No. LL202580, (2025)). Written informed consent was waived by the ethics committee due to the retrospective nature of the study, with all data anonymized to protect patient privacy. All procedures were performed in accordance with the Declaration of Helsinki.

**Figure 1:**
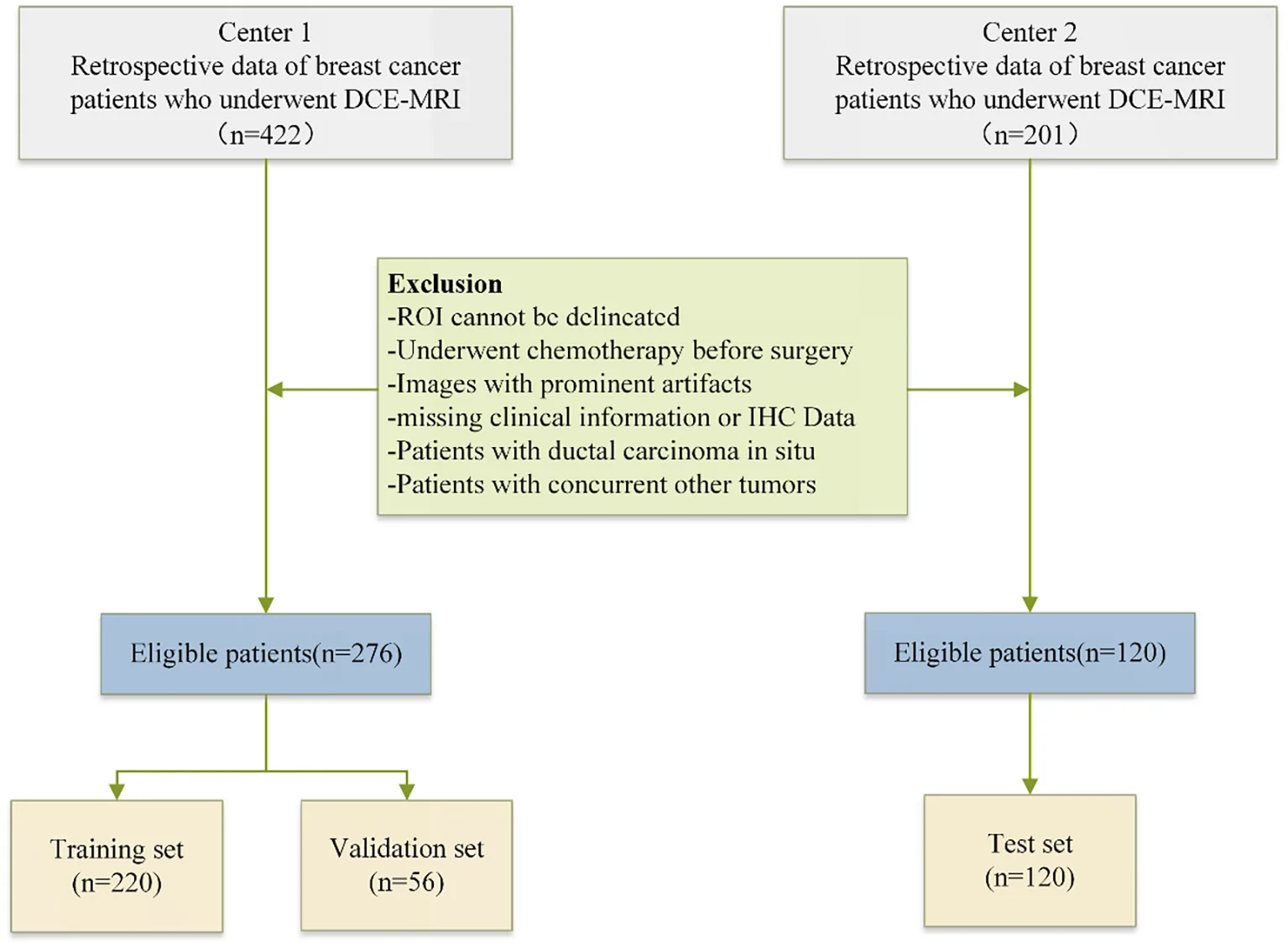
The flowchart of inclusion and exclusion criteria.

Consecutive patients who underwent surgery and were pathologically confirmed as invasive breast cancer between January 2021 and October 2025 were enrolled from two independent medical institutions. Patients were included if they met all of the following criteria: (1) histologically verified invasive breast cancer; (2) DCE-MRI examination completed within 2 weeks before surgery; (3) complete immunohistochemical results of ER, PR, HER2 and Ki-67 available for molecular subtyping; (4) no prior neoadjuvant therapy or breast surgery; (5) DCE-MRI images with acceptable quality and no severe artifacts. Patients were excluded if: (1) they had non-invasive lesions such as ductal carcinoma *in situ*; (2) clinical, imaging or pathological data were incomplete; (3) the tumor was too scattered to be manually segmented; (4) other malignant tumors were present. The flowchart of patient inclusion and exclusion criteria is shown in **Figure 1**.

Patients in Center A were randomly divided into a training cohort and an internal validation cohort at a ratio of 8:2. Patients from Center B served as an independent external test cohort. The training cohort was used for model fitting, the internal validation cohort for model selection and hyperparameter tuning, and the external test cohort exclusively for final performance evaluation, ensuring the model’s generalization ability across different clinical centers. Clinical features including age, menstrual status, lymph node status, T stage, WHO grade and IHC indicators were collected. According to IHC results, patients were classified into luminal subtype (Luminal A/B) and non-luminal subtype (HER2-enriched/triple-negative).

### 2.2 MRI acquisition

Center A used a 3.0T (Achieva, Philips, Amsterdam, the Netherlands) and a 1.5T (MAGNETOM Amira, Siemens Healthcare, Erlangen, Germany) MRI scanner. Center B used a Siemens 3.0T (MAGNETOM Verio, Siemens Healthcare, Erlangen, Germany) MRI scanner. All patients were positioned prone with both breasts hanging naturally in a dedicated breast coil. Routine sequences included axial T1WI without fat saturation, axial T2WI with fat saturation, axial DWI, and axial fat-suppressed T1WI dynamic contrast-enhanced scanning. Detailed scanning parameters are shown in **Supplementary Table 1**. Gadolinium-DTPA (Magnevist, Schering, Germany) was injected intravenously at a dose of 0.1 mmol/kg, followed by a 20 mL saline flush.

### 2.3 Image preprocessing and segmentation

The second phase of DCE-MRI images was exported from the PACS system for analysis. Image preprocessing and ROI segmentation were performed using 3D Slicer (version 5.0.3) and Python (version 3.11.4). Three standard preprocessing operations were performed: (1) trilinear interpolation to resample voxels to 1 mm × 1 mm × 1 mm; (2) N4 bias field correction to reduce signal inhomogeneity; (3) Z-score normalization to unify the intensity distribution. Two radiologists with 8 and 10 years of experience in breast MRI independently manually segmented tumor ROIs slice by slice to cover the whole tumor. Fifty cases were randomly selected to evaluate intra-observer and inter-observer consistency using intraclass correlation coefficient (ICC) and Dice similarity coefficient (DSC).

### 2.4 Habitat imaging

**Figure 2:**
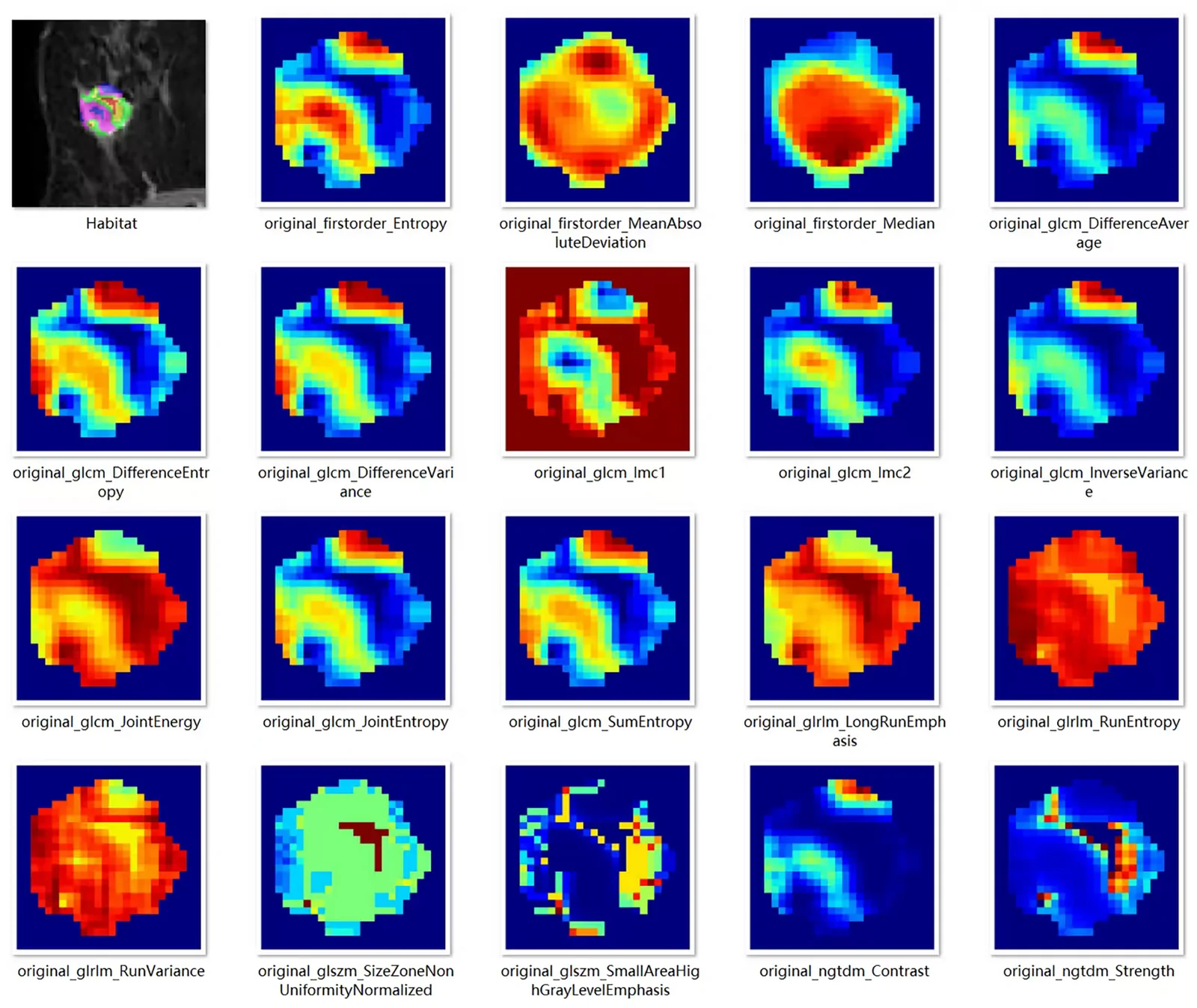
Habitat regions generation and characteristics visualization.

Tumor habitat subdivision was achieved through a two-step strategy combining Simple Linear Iterative Clustering (SLIC) (13) superpixel segmentation and K-means clustering (14). First, the preprocessed DCE-MRI tumor ROI was smoothed using a 3×3×3 Gaussian kernel to reduce noise. SLIC superpixel segmentation was performed with 100 superpixels, compactness of 10, and gradient threshold of 50 to avoid edge interference. The maximum iteration was set to 10, and isolated superpixels smaller than 5 voxels were merged into adjacent regions. A 3×3×3 sliding window was used to extract local features, and a 19-dimensional feature vector was generated for each voxel (**Figure 2**).

**Figure 3:**
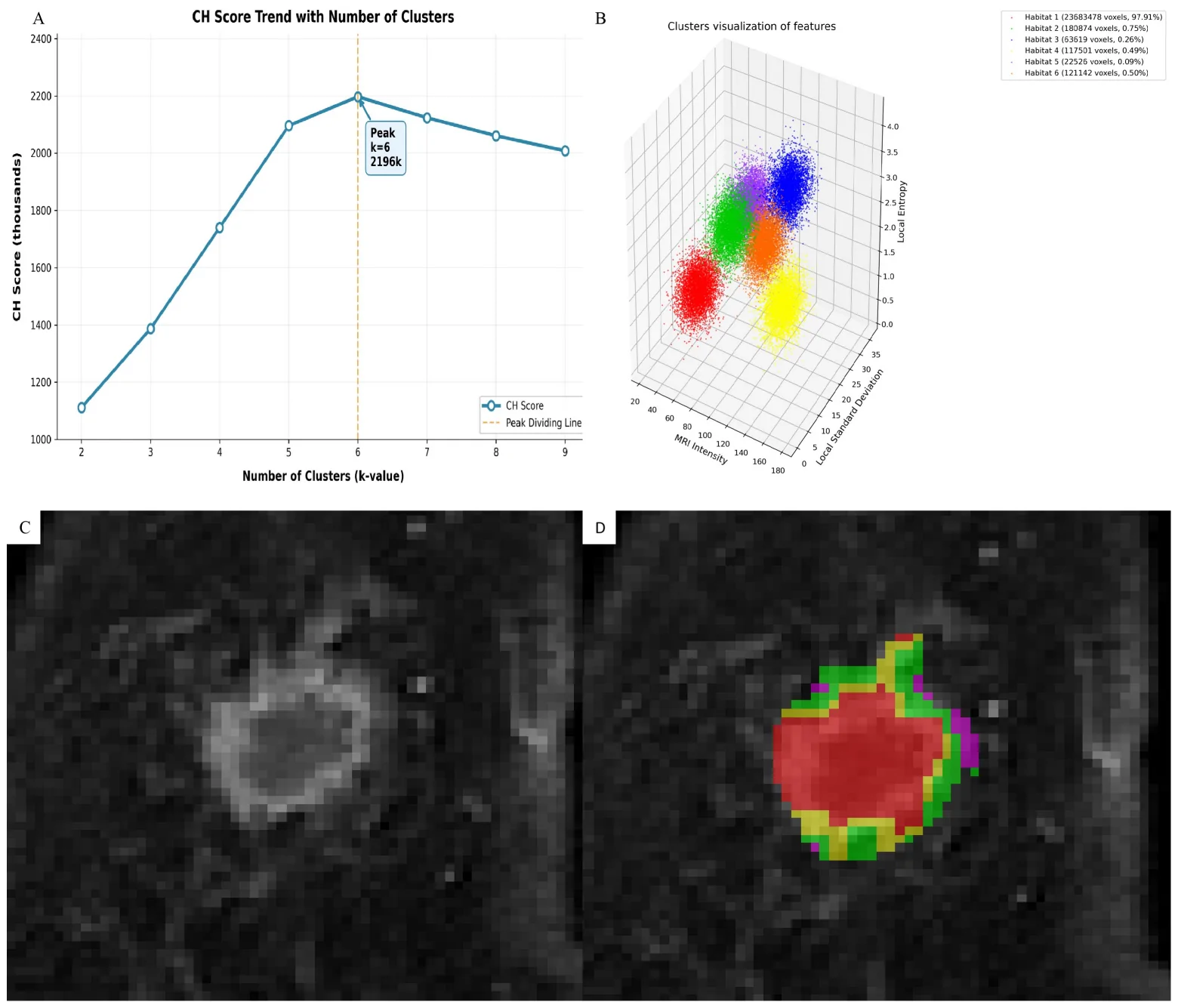
Habitat clustering results. **(A)** Calinski–Harabasz score line chart. **(B)** three-dimensional visualization of Clusters. **(C)** Dynamic contrast-enhanced magnetic resonance imaging. **(D)** Habitat imaging.

K-means clustering was performed based on these feature vectors using Euclidean distance as the similarity measure. The optimal cluster number was determined using the Calinski–Harabasz (CH) (15) index in the range of k=2 to 9. The CH index reached its maximum value at k=6 (**Figures 3A, B**), indicating the optimal trade-off between inter-cluster separation and intra-cluster compactness. Clustering stopped when the maximum iteration reached 300 or the cluster center shift was less than 10^-4^. Clusters accounting for less than 2% of all superpixels were regarded as noise and merged into the most similar subregion. Finally, six habitat subregions were mapped to the original image to generate the habitat segmentation map (**Figures 3C, D**).

### 2.5 Radiomics feature extraction and selection

We conducted feature extraction using the Pyradiomics (version 3.0.1) following the Imaging Biomarker Standardization Initiative guidelines. Two categories of radiomics features were generated for subsequent analyses. For traditional radiomics features, a total of one hundred and seven manually curated radiomics features, covering shape descriptors, first-order statistics, and texture features, were extracted from the entire tumor ROI. In contrast, the workflow for habitat radiomics features involved a two-step process: radiomics features were separately extracted from each predefined habitat sub-region, and these sub-region-specific features were then subjected to feature fusion to derive the final habitat features.

Feature selection was performed sequentially. Significant features with P < 0.05 were retained by t-test or Mann–Whitney U test. Redundant features with correlation coefficient above 0.9 were removed using Pearson analysis. Key features with non-zero coefficients were finally selected by least absolute shrinkage and selection operator (LASSO) regression integrated with 10-fold cross-validation for model construction. Feature selection was carried out exclusively on the training set to prevent information leakage.

### 2.6 Model development

Four predictive models were constructed: clinical model, conventional radiomics model, habitat radiomics model, and stacking [post-fusion (16)] model. For the clinical model, univariate and multivariate logistic regression were used to screen independent predictors. Missing values were filled by median, and outliers were corrected using the interquartile range (IQR) method.

In parallel, eight machine learning algorithms [Random Forest (RF), eXtreme Gradient Boosting (XGBoost), Light Gradient Boosting Machine (LGBM), Extra Trees (ET), Adaptive Boosting (AdaBoost), Multi-Layer Perceptron (MLP), Support Vector Machine (SVM), and Logistic Regression (LR)] were trained on three feature sets (eligible clinical features, LASSO-reduced traditional radiomics features, habitat imaging features). Hyperparameter tuning and optimization for all models were performed exclusively on the training set using 10-fold cross-validation, and a grid search algorithm was adopted to traverse parameter combinations and determine the optimal hyperparameter configuration for each model. The final optimal model was selected according to the AUC performance on the internal validation set (17).

**Figure 4:**
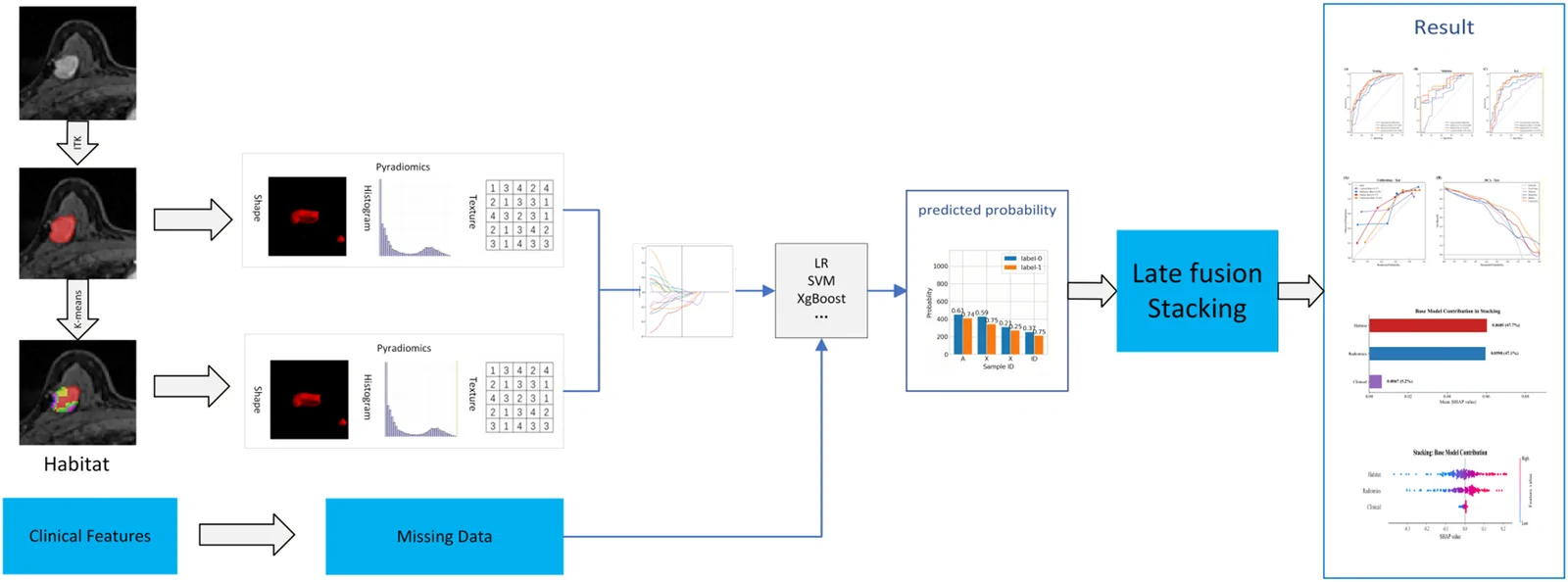
Workflow of this study.

The stacking model employed three top-performing base learners and a logistic regression meta-learner with favorable interpretability and stable generalization performance to construct a two-layer ensemble framework. To eliminate data leakage and optimistic bias, 10-fold stratified cross-validation with out-of-fold (OOF) prediction was adopted to generate meta-features. The aggregated OOF probabilities were exclusively used for meta-learner training instead of direct full-training fitting results. All base models were retrained on the complete training set and weight-frozen for stable forward inference. All training, feature generation and parameter tuning were restricted to the training set. The internal validation set guided structural optimization and preliminary performance evaluation, while the external test set remained entirely unseen throughout model development and was only used for final independent generalization validation. **Figure 4** illustrates the core model construction workflow.

### 2.7 Statistical analysis

Statistical analyses were performed using Python 3.9.7 and R 4.2.1. Baseline characteristics were compared using the Student’s t-test or Mann-Whitney U test for continuous variables, and the chi-square test for categorical variables. Variance inflation factor (VIF) was calculated to assess multicollinearity across clinical predictors. Model calibration was assessed with calibration curves. Model predictive performance was evaluated using the area under the curve (AUC), accuracy, sensitivity, and specificity. Optimal cutoffs were derived from training-set Youden’s J index and fixed for validation and test sets to avoid data leakage. SHapley Additive exPlanations (SHAP) analysis was applied to assess feature contributions and improve model interpretability. Decision curve analysis (DCA) was conducted to quantify the net clinical benefit. A two-tailed p-value < 0.05 was considered statistically significant.

## 3 Results

### 3.1 Clinical data

The clinical features of patients are summarized in **Table 1**. A total of 396 patients (265 luminal patients and 131 non-luminal patients) were enrolled and divided into the training set (n=220), internal validation set (n=56), and external test set(n=120). Baseline analyses showed no significant differences in continuous variables (age, Ki-67) among sets (all p>0.05). For categorical variables, menopausal status was comparable (p>0.05), but significant variations in T stage, LN status, HER2 expression, and WHO grade (all p<0.05)—the training and validation sets had higher LN metastasis, and a higher proportion of HER2 expression and T2 stage patients in the external test set.

**Table 1:** Clinical characteristics of the training set, validation and test set.

Variables	All (n=396)	Training set (n=220)	Validation set (n=56)	Test set (n=120)	P value
Age (year), mean±SD	50.37±9.78	50.93±10.02	48.89±10.00	50.03±9.23	0.343
Ki67, mean±SD	0.33±0.19	0.34±0.21	0.32±0.18	0.36±0.21	0.408
Menopausal status, n (%)					0.437
Pre-Menopausal	209 (52.78)	113 (51.36)	34 (60.71)	62 (51.67)	
Post-Menopausal	187 (47.22)	107 (48.64)	22 (39.29)	58 (48.33)	
LN Metastasis, n (%)					<0.001*
No metastasis	221 (55.81)	106 (48.18)	31 (55.36)	84 (70.00)	
Metastasis	175 (44.19)	114 (51.82)	25 (44.64)	36 (30.00)	
HER2, n (%)					0.015*
Negative	239 (60.35)	140 (63.64)	39 (69.64)	60 (50.00)	
Positive	157 (39.65)	80 (36.36)	17 (30.36)	60 (50.00)	
WHO grade, n (%)					0.004*
1	31 (7.83)	25 (11.36)	3 (5.36)	3 (2.50)	
2	281 (70.96)	159 (72.27)	41 (73.21)	81 (67.50)	
3	84 (21.21)	36 (16.36)	12 (21.43)	36 (30.00)	
T stage, n (%)					0.001*
1	143 (36.11)	95 (43.18)	17 (30.36)	31 (25.83)	
2	217 (54.80)	104 (47.27)	30 (53.57)	83 (69.17)	
3	27 (6.82)	15 (6.82)	6 (10.71)	6 (5.00)	
4	9 (2.27)	6 (2.73)	3 (5.36)		
Molecular subtype, n (%)					0.087
Luminal	265 (66.92)	136 (61.82)	42 (75.00)	87 (72.50)	
Non-luminal	131 (33.08)	84 (38.18)	14 (25.00)	33 (27.50)	

Data are presented as n (%) or mean ± standard deviation. LN: lymph node; HER2: human epidermal growth factor receptor 2; WHO: World Health Organization.

*p<0.05.

All VIF values were less than 5 revealing no multicollinearity (**Supplementary Table 2**). Univariate and multivariate logistic regression analyses results are summarized in **Table 2**. In the univariate analysis, age (odds ratio [OR] = 1.009, P = 0.001), WHO histological grade (OR = 1.201, P = 0.005), T stage (OR = 1.233, P = 0.006), and LN status (OR = 1.478, P = 0.041) were significantly correlated with the target outcome; after multivariate adjustment, only age (OR = 1.02, P = 0.031) remained statistically associated with the target outcome, whereas no significant correlations were observed for the other features (P > 0.05).

**Table 2:** Univariate and multivariate logistic regression analysis.

Variables	Univariate analysis		Multivariate analysis	
	OR (95%CI)	P value	OR (95%CI)	P value
HER2	0.667(0.458-2.970)	0.076		
age	1.009(1.005-1.013)	0.001*	1.020(1.005-1.035)	0.031*
Ki-67	1.012(0.992-1.034)	0.328		
WHO	1.201(1.079-1.338)	0.005*	0.782(0.536-1.141)	0.285
T stage	1.233(1.089-1.397)	0.006*	0.972(0.697-1.357)	0.890
Menopausal status	1.432(0.937-1.978)	0.068		
LN	1.478 (1.080-2.024)	0.041*	0.992(0.607-1.623)	0.979

LN, lymph node; HER2, human epidermal growth factor receptor 2; WHO, World Health Organization; OR, odds ratio; CI, confidence interval.

*p<0.05.

During the process of building the clinic model, we compared eight different algorithms and found that the XGBoost exhibited the best performance among them (**Supplementary Table 3**). Specifically, on the training set, the accuracy, sensitivity, specificity, and AUC were 0.741, 0.809, 0.631, and 0.760 (95% confidence interval [CI]: 0.694 - 0.822), respectively; on the validation set, these metrics were 0.589, 0.595, 0.571, and 0.650 (95% CI: 0.460 - 0.825), respectively; and on the external testing set, the corresponding values were 0.658, 0.678, 0.606, and 0.652 (95% CI: 0.535 - 0.764).

### 3.2 Inter- and intra-observer agreements

Fifty cases were randomly selected to evaluate both intra-observer and inter-observer segmentation consistency. For intra-observer evaluation, one radiologist repeated the manual segmentation after an interval of two weeks to avoid memory bias. The two-way random-effects intraclass correlation coefficient (ICC) and Dice similarity coefficient (DSC) were calculated based on tumor volume. The inter-observer ICC was 0.88 (DSC = 0.85), and the intra-observer ICC was 0.92 (DSC = 0.89).

### 3.3 Traditional radiomics model

**Figure 5:**
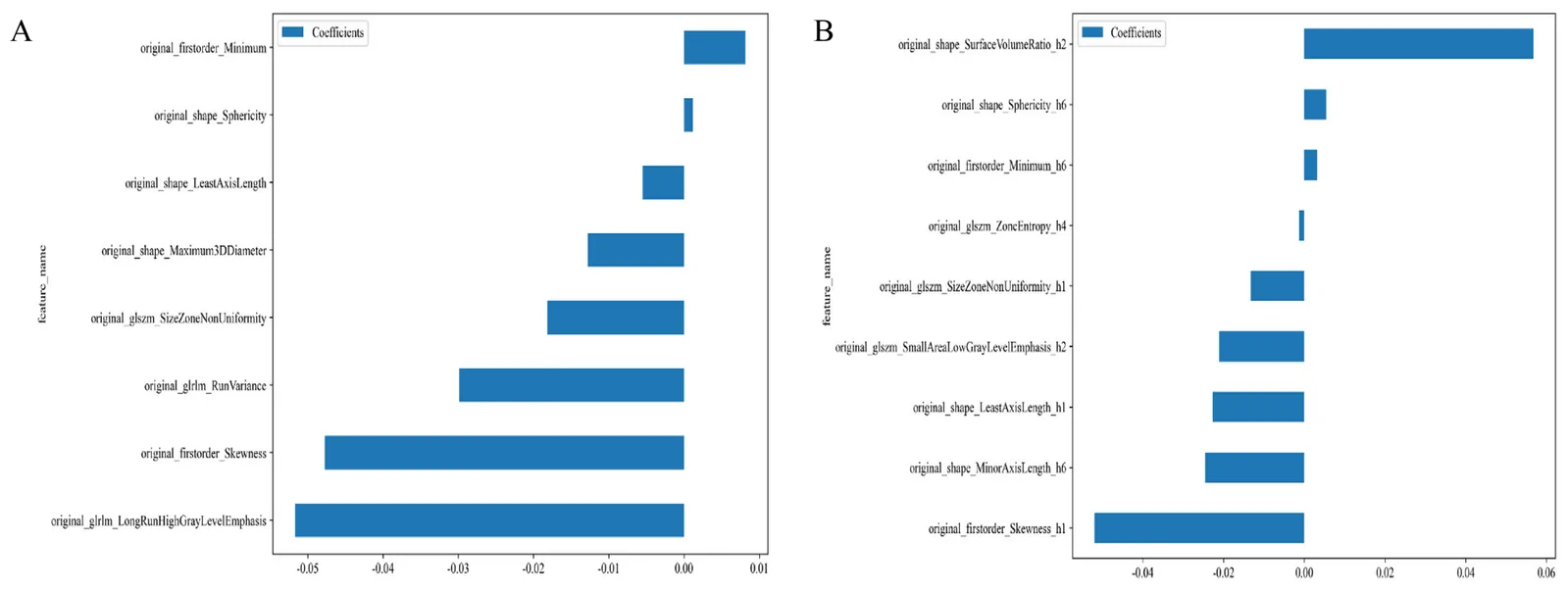
SHAP Analysis for interpretation of the Stacking model. **(A)** SHAP summary plot, **(B)** feature importance. SHAP, SHapley Additive exPlanations.

For conventional radiomic analysis, a total of 107 features were extracted from the entire segmented tumor region, encompassing 18 first-order features, 24 gray-level co-occurrence matrix (GLCM) features, 14 gray-level dependence matrix (GLDM) features, 16 gray-level run length matrix (GLRLM) features, 16 gray-level size zone matrix (GLSZM) features, 5 neighboring gray tone difference matrix (NGTDM) features, and 14 shape features. Following dimensionality reduction with the Lasso algorithm, 8 features were ultimately retained and subsequently integrated into machine learning model construction (**Figure 5A**). During the process of building the machine learning model, the LGBM exhibited the best performance (**Supplementary Table 4**). Specifically, on the training set, the accuracy, sensitivity, specificity, and AUC were 0.750, 0.897, 0.512, and 0.802 (95% CI: 0.741 - 0.860), respectively; on the validation set, these metrics were 0.750, 0.857, 0.429, and 0.774 (95% CI: 0.646 - 0.898), respectively; and on the external testing set, the corresponding values were 0.758, 0.736, 0.718, and 0.805 (95% CI: 0.718 - 0.884).

### 3.4 Habitat radiomics model

A total of 642 features were extracted from the 6 clusters and integrated to build a comprehensive tumor microenvironment signature. Subsequent dimensionality reduction via the Lasso algorithm ultimately retained 9 core features (**Figure 5B**), which were then used for subsequent machine learning model construction. The LGBM stood out with its best performance (**Supplementary Table 5**). Specifically, on the training set, the accuracy, sensitivity, specificity, and AUC were 0.786, 0.904, 0.595, and 0.854 (95% CI: 0.803 - 0.904), respectively; on the validation set, these metrics were 0.786, 0.881, 0.500, and 0.830 (95% CI: 0.713 - 0.932), respectively; and on the external testing set, the corresponding values were 0.742, 0.736, 0.758, and 0.830 (95% CI: 0.727 - 0.916).

### 3.5 Stacking model

Taking habitat-LGBM, traditional radiomics-LGBM and clinical-XGBoost as base learners and logistic regression as meta-classifier, a stacking model was constructed.

Specifically, on the training set, the accuracy, sensitivity, specificity, and AUC were 0.768, 0.949, 0.676, and 0.863 (95% CI: 0.811 - 0.909), respectively; on the validation set, these metrics were 0.786, 0.905, 0.629, and 0.849 (95% CI: 0.742 - 0.941), respectively; and on the external testing set, the corresponding values were 0.800, 0.816, 0.758, and 0.840 (95% CI: 0.751 - 0.921).

### 3.6 Performance and comparison of different models

**Figure 6:**
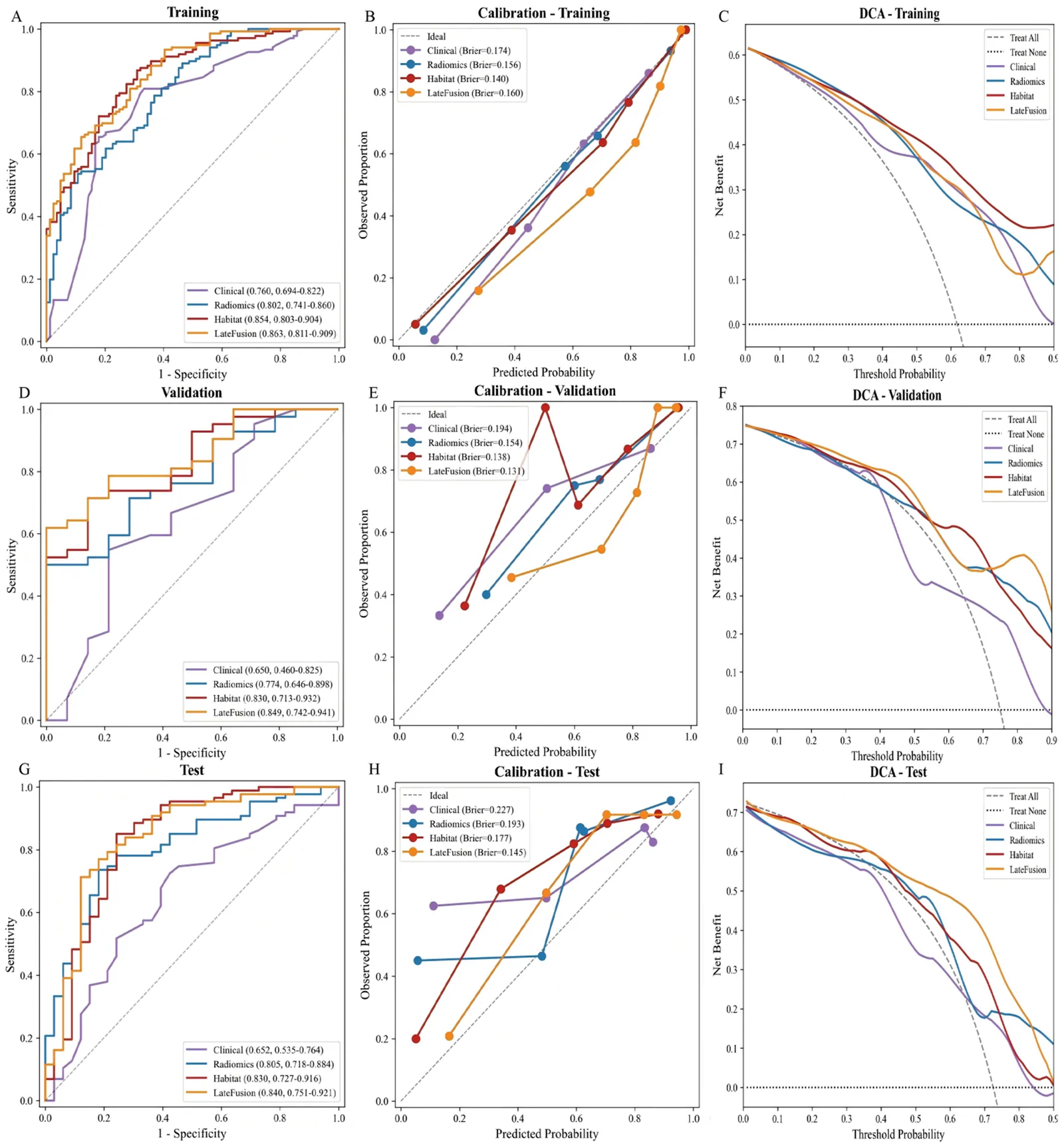
The performance of habitat-LGBM, traditional radiomics-LGBM, clinical-XGBoost and Stacking model in the training set, validation and test sets. The AUC, calibration curve and DCA of models in the training set **(A–C)**, validation set **(D–F)** and test set **(G–I)**. AUC, area under the receiver operating characteristic curve; DCA, decision curve analysis.

**Figure 7:**
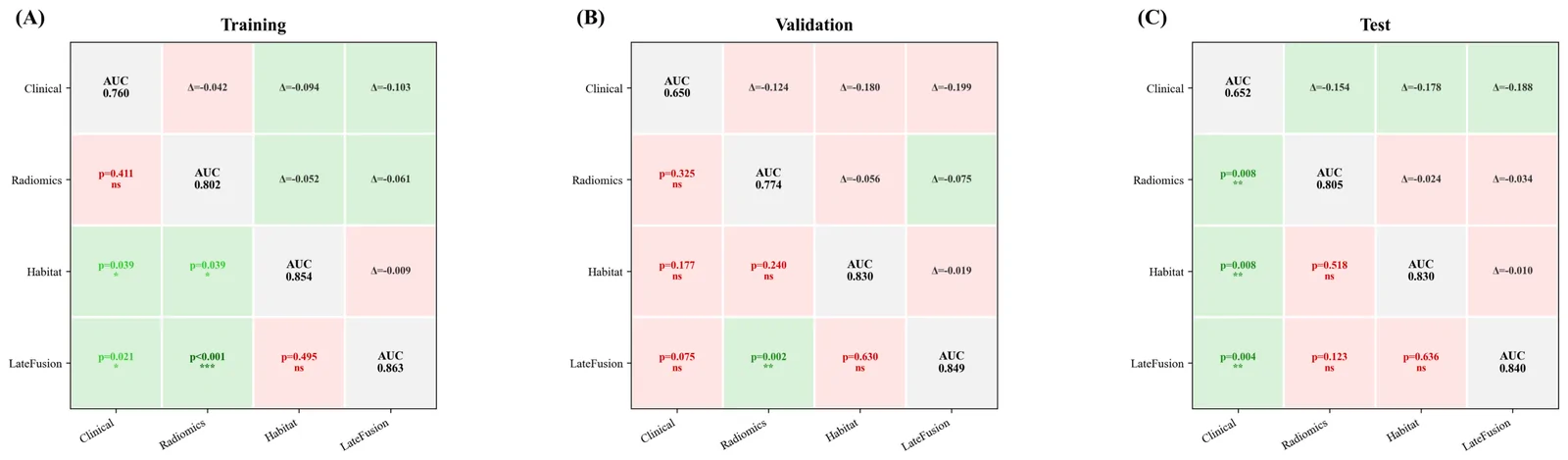
DeLong test for pairwise comparisons among four different models. **(A–C)** represent pairwise comparisons of the four models in the training, validation, and test datasets, respectively. Statistically significant results are shown in green with asterisks, and non-significant ones in red, with AUC values highlighted along the diagonal for each set.

Summary of predictive performance for four different models was presented in **Table 3** and **Figure 6**. DeLong testing of AUC among four different models presented in **Figure 7**. For the discrimination of breast cancer luminal and non-luminal subtypes, the stacking model yielded the largest area under the curve value (AUC = 0.840), followed by the habitat model and the conventional radiomics model (AUC = 0.830, AUC = 0.805, respectively), all of which were significantly better than the clinical model (p < 0.05, respectively).

**Table 3:** Performances of the predictive models in the training set, validation and test set.

Model and metric	AUC	95%CI	Accuracy	sensitivity	Specificity
Training set (n=220)					
Radiomics-LGBM	0.802	0.741 - 0.860	0.750	0.897	0.512
Habitat-LGBM	0.854	0.803 - 0.904	0.786	0.904	0.595
Clinic-XGBoost	0.760	0.694 - 0.822	0.741	0.809	0.631
Stacking	0.863	0.811 - 0.909	0.768	0.949	0.676
Validation set (n=56)					
Radiomics-LGBM	0.774	0.646 - 0.898	0.750	0.857	0.429
Habitat-LGBM	0.830	0.713 - 0.932	0.786	0.881	0.500
Clinic-XGBoost	0.650	0.460 - 0.825	0.589	0.595	0.571
Stacking	0.849	0.742 - 0.941	0.786	0.905	0.629
Test set (n=120)					
Radiomics-LGBM	0.805	0.718 - 0.884	0.758	0.736	0.718
Habitat-LGBM	0.830	0.727 - 0.916	0.742	0.736	0.758
Clinic-XGBoost	0.652	0.535 - 0.764	0.658	0.678	0.606
Stacking	0.840	0.751 - 0.921	0.800	0.816	0.758

AUC, area under the receiver operating characteristic curve; CI, confidence interval; LGBM, light gradient boosting machine; XGBoost, extreme gradient boost.

**Figure 8:**
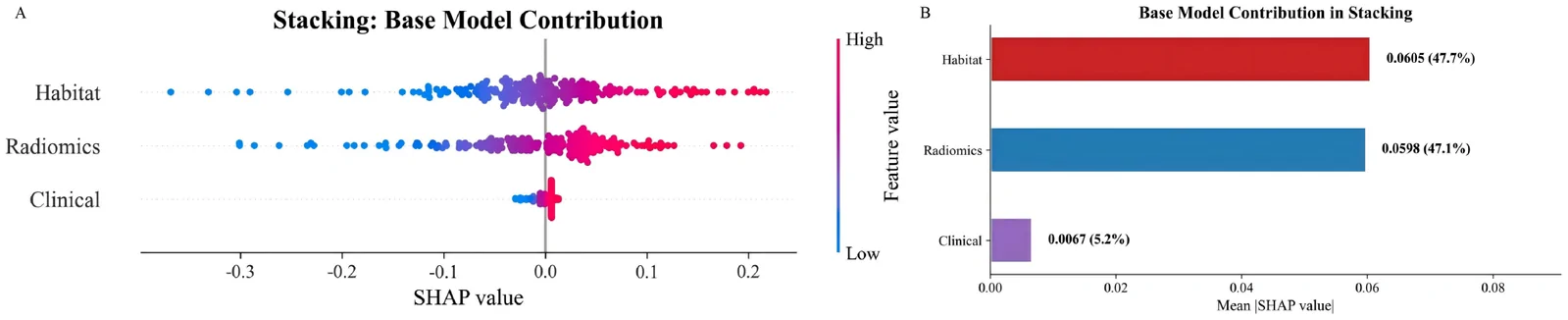
Core features of radiomics model. **(A)** core features of traditional radiomics model. **(B)** core features of Habitat Radiomics Model.

The stacking model showed the lowest Brier score (0.145) among on the external test set, with its calibration curve lying closest to the diagonal of ideal calibration. Decision curve analysis confirmed that the stacking model achieved the highest net benefit across the majority of risk threshold probabilities, relative to the other predictive models as well as the treat-all and treat-none reference strategies. The SHAP plot intuitively revealed the core and significant role of habitat-LGBM model, with their contribution being particularly prominent (**Figure 8**).

## 4 Discussion

Accurate differentiation between luminal and non-luminal subtypes in invasive breast cancer is critical for survival and personalized treatment. This study presents a DCE-MRI-based radiomics strategy integrating clinical features, conventional radiomic features, and six-subregion habitat features. A novel stacking model was constructed for efficient subtype classification, showing great potential in guiding personalized therapy. As the core component within this stacking framework, six-subregion habitat analysis exhibits the potential to further enhance predictive efficacy and net benefit.

Age, Ki-67 expression, and menopausal status were well balanced across the three cohorts (p>0.05), ensuring stable model training and valid internal validation. Nevertheless, the cohorts differed in several key baseline clinicopathological features. Such real-world covariate imbalances are an inherent limitation of the present study and may partially impair the external generalization of purely clinical prediction models. While univariate analysis identified multiple clinicopathological factors associated with Luminal subtype prediction, only age remained an independent predictor after multivariate correction, which aligns with the modest external predictive efficacy of the clinical model (AUC = 0.652). Traditional macroscopic clinical indicators are prone to the impacts of population heterogeneity and subjective assessment bias, resulting in insufficient robustness across different cohorts. The distribution discrepancy between training and external validation cohorts weakens the model’s discriminative capability and degrades its predictive performance. Importantly, such inter-cohort heterogeneity objectively reflects real-world clinical conditions, enabling rigorous external validation and preventing overoptimistic outcomes caused by homogeneous cohort data. In contrast to conventional clinical variables, radiomic features can capture intratumoral microscopic heterogeneity and are less affected by baseline variations. Accordingly, incorporating radiomic signatures can effectively compensate for the deficiencies of pure clinical models and enhance their generalization performance in practical clinical applications (18).

DCE-MRI provides images with high time, high space and high signal-to-noise ratio for the diagnosis of breast lesions by evaluating tumor morphology and hemodynamics (19). The images in the second phase were obviously enhanced to better reflect the aggressiveness and heterogeneity of the tumor (20). Consistent with our research results, previous studies have successfully used traditional radiomics models based on DCE-MRI in determination of molecular subtypes of breast cancer (21–23). However, these studies did not deeply analyze the heterogeneity within tumors. Several recent studies have shown the potential clinical application of radiomics as a promising biomarker through habitat imaging analysis (24–27). Based on the above research background, SLIC coupled with K-means clustering, a widely validated pipeline for tumor habitat analysis across multiple cancers (26, 27), was utilized in this study to segment intratumoral habitats, and we investigated whether habitat imaging could distinguish breast cancer molecular subtypes. Our results showed that, across the training set, validation set, and external test set (AUC = 0.854 vs 0.802, 0.830 vs 0.774, and 0.830 vs 0.805), the Habitat-LGBM model demonstrated a trend toward higher AUC values compared with the traditional radiomics-LGBM model. Habitat imaging discerns and leverages heterogeneous information conveyed by distinct subregions through tumor region re-segmentation, thereby shedding light on the underlying reason why habitat-based models outperform traditional radiomics approaches (28).

Notably, our approach integrates habitat-LGBM, traditional radiomics-LGBM, and clinical-XGBoost to develop a stacking model. Stacking—a robust ensemble learning technique—integrates the outputs (prediction probabilities) of multiple base models through a meta-classifier to generate a unified predictive result, thereby constructing a post-fusion framework that fully capitalizes on the unique strengths of each individual model and further improves overall classification performance. Li et al (29) reported that their late fusion model achieved the highest AUC and outperformed the early fusion model in predicting occult lymph node metastasis in tongue squamous cell carcinoma. Similarly, a stacking model combining habitat analysis and the Vision Transformer (ViT) model, developed based on high-resolution vessel wall imaging, exhibited superior performance in identifying high-risk vulnerable plaques in patients with symptomatic intracranial atherosclerotic stenosis and predicting the risk of stroke recurrence (30). Tian et al (31) further proposed a stacking model integrating digital breast tomosynthesis (DBT) craniocaudal projections and ultrasound images, which yielded the optimal classification performance for distinguishing benign from malignant breast tumors. In our study, the proposed stacking model achieved the numerically highest AUC value, with excellent specificity and high sensitivity. Calibration curves verified consistent predictive calibration, and decision curve analysis validated its favorable net clinical benefit. SHAP plots intuitively illustrated the core and pivotal role of the habitat-LGBM model within the stacking framework, with its contribution being particularly prominent—this further validates the critical importance of habitat imaging analysis in our research.

Enhancements in computational capabilities and algorithmic refinements have propelled the substantial headway of machine learning in prognosticating ailments (32, 33). XGBoost and LGBM excel in optimizing both speed and accuracy when processing structured data (34). Previous research (35) demonstrated that an XGBoost model derived from multi-parametric MRI holds promise as a decision-support tool for the preoperative identification of luminal and non-luminal breast cancer subtypes. Notably, their study compared five distinct machine learning models, yet LGBM was not included. In contrast, our research constructed eight machine learning predictive models to distinguish between breast cancer patients with different molecular subtypes—XGBoost exhibited optimal performance in the clinical model, while LGBM outperformed other approaches in the radiomics-based framework. As a gradient-boosting framework rooted in tree-based learning algorithms, LGBM effectively captures the nonlinear correlations among radiomics features (36). This attribute is particularly well-suited for identifying tumor heterogeneity and microenvironmental changes on MRI, thereby enhancing its utility for predicting breast cancer subtypes. Furthermore, this finding underscores that integrating diverse machine learning models may represent a more comprehensive and effective strategy.

These results point to a possible clinical use. On the external test set, the stacking model classified luminal versus non-luminal cases with an AUC of 0.840. Luminal tumors are hormone-receptor positive and generally respond well to endocrine therapy. A model that integrates imaging and clinical features to characterize luminal status could serve as a complementary tool to support molecular subtyping, particularly when immunohistochemistry is inconclusive or biopsy sampling is limited by intratumoral heterogeneity. As an imaging-based aid, it is intended to complement rather than replace standard pathological assessment.

This study has several notable limitations. First, it adopts a retrospective design, which carries inherent potential selection bias and cannot fully eliminate confounding clinical factors. Second, the external validation cohort has a relatively small sample size, and the three datasets exhibit imbalanced baseline characteristics, which may affect the stability and reproducibility of model performance. Third, MRI data involves heterogeneous scanner models, magnetic field strengths and scanning protocols, introducing inherent imaging acquisition variability. Fourth, the model lacks validation on prospective independent cohorts, and its overall external generalizability remains limited. Further validation using large, balanced, prospective multicenter cohorts is necessary to confirm model robustness.

## 5 Conclusions

DCE-MRI-based habitat radiomics may assist in differentiating breast cancer molecular subtypes. The stacking model achieves feasible discrimination between luminal and non-luminal subtypes and offers a new evaluation strategy, yet further research is needed before clinical use.

## Data Availability

The original contributions presented in the study are included in the article/**Supplementary Material**. Further inquiries can be directed to the corresponding author.
