# Supplementary material for: Habitat imaging based on DCE-MRI for differentiating luminal and non-luminal subtypes of breast cancer: a two-center study
Source: Front Oncol. 2026 Sep 7;16:1899349. doi: 10.3389/fonc.2026.1899349 (PMC13590339; doi:10.3389/fonc.2026.1899349)
Supplement: Supplementary file 1 [file Table1.pdf]

Supplementary Table S1: Parameter details of MRI Acquisition

Parameters	Center1(MR1)	Center1(MR2)	Center2(MR3)
Scanner Model	Siemens MAGNETOM Amira 1.5T	Philips Achieva 3.0T	Siemens MAGNETOM Verio 3.0T
TR(ms)	4.95	4.8	4.2
TE(ms)	2.39	2.4	1.9
Flip Angle	10	10	12
NEX	1	1	1
FOV(mm)	340×340	340×340	320×320
Acquisition Matrix	345×269	340×340	320×320
Reconstruction Matrix	345×269	352×352	512×512
Slice Thickness (Acquisition, mm)	1.6	1	1.5
Slice Thickness (Reconstruction, mm)	1.6	1	0.75
Slice Overlap (mm)	None	None	0.75
Voxel Size (mm^3)	0.4×0.4×1.6	1.0×1.0×1.0	0.625×0.625×0.75
Phase Configuration	9	9	8
Single-phase Duration (s)	73	61	60
Total Scanning Duration (s)	660	556	480
Contrast Agent Dosage (mL/kg)	0.2	0.2	0.1
Contrast Agent Injection Rate (mL/s)	2.5	2.5	2.5

TR: Repetition Time; TE: Echo Time; FOV: Field of View; NEX: Number of Excitations

Supplementary Table S2: Variance inflation factors (VIF) of clinical predictors

Variables	VIF
HER2	1.51
age	2.26
Ki-67	1.28
WHO	1.75
T stage	1.67
Menopausal status	2.43
LN	1.49

HER2: human epidermal growth factor receptor 2; WHO: World Health Organization; LN: lymph node; VIF: variance inflation factor.

Supplementary Table S3: Performances of the diverse machine learning predictive models of Clinical data in the training set

model_name	Accuracy	AUC	95% CI	Sensitivity	Specificity
LR	0.723	0.714	0.675 - 0.762	0.754	0.512
SVM	0.618	0.534	0.468 - 0.685	0.667	0.418
RF	0.796	0.703	0.648 - 0.836	0.831	0.555
ET	0.723	0.747	0.681 - 0.775	0.754	0.624
XGBoost	0.741	0.760	0.694 - 0.822	0.809	0.631
LGBM	0.749	0.723	0.654 - 0.756	0.734	0.642
AdaBoost	0.709	0.689	0.572 - 0.786	0.809	0.578
MLP	0.624	0.642	0.608 - 0.695	0.623	0.512

AUC: area under the receiver operating characteristic curve; CI: confidence interval; LR: Logistic Regression; SVM: Support Vector Machine; RF: Random Forest; ET: Extra Trees; XGBoost: eXtreme Gradient Boosting; LGBM: Light Gradient Boosting Machine; Adaboost: Adaptive Boosting; MLP: Multi-Layer Perceptron.

Supplementary Table S4: Performances of the diverse machine learning predictive models of traditional radiomics in the training set

model_name	Accuracy	AUC	95% CI	Sensitivity	Specificity
LR	0.664	0.657	0.582 - 0.732	0.765	0.500
SVM	0.632	0.612	0.533 - 0.691	0.743	0.452
RF	0.773	0.785	0.720 - 0.849	0.831	0.679
ET	0.641	0.691	0.618 - 0.763	0.603	0.702
XGBoost	0.695	0.772	0.711 - 0.834	0.669	0.738
LGBM	0.750	0.802	0.741 - 0.860	0.897	0.512
AdaBoost	0.709	0.793	0.719 - 0.877	0.858	0.655
MLP	0.650	0.661	0.587 - 0.735	0.699	0.571

AUC: area under the receiver operating characteristic curve; CI: confidence interval; LR: Logistic Regression; SVM: Support Vector Machine; RF: Random Forest; ET: Extra Trees; XGBoost: eXtreme Gradient Boosting; LGBM: Light Gradient Boosting Machine; Adaboost: Adaptive Boosting; MLP: Multi-Layer Perceptron.

Supplementary Table S5: Performances of the diverse machine learning predictive models of habitat radiomics in the training set

model_name	Accuracy	AUC	95% CI	Sensitivity	Specificity
LR	0.673	0.702	0.632 - 0.772	0.728	0.583
SVM	0.591	0.563	0.482 - 0.644	0.603	0.571
RF	0.718	0.792	0.731 - 0.853	0.662	0.810
ET	0.609	0.694	0.624 - 0.764	0.529	0.738
XGBoost	0.750	0.826	0.732 - 0.908	0.776	0.669
LGBM	0.786	0.854	0.803 - 0.904	0.904	0.595
AdaBoost	0.773	0.832	0.779 - 0.884	0.779	0.762
MLP	0.782	0.834	0.782 - 0.887	0.831	0.702

AUC: area under the receiver operating characteristic curve; CI: confidence interval; LR: Logistic Regression; SVM: Support Vector Machine; RF: Random Forest; ET: Extra Trees; XGBoost: eXtreme Gradient Boosting; LGBM: Light Gradient Boosting Machine; Adaboost: Adaptive Boosting; MLP: Multi-Layer Perceptron.
